# Measuring and Quantifying Collateral Information in Psychiatry: Development and Preliminary Validation of the McLean Collateral Information and Clinical Actionability Scale

**DOI:** 10.2196/25050

**Published:** 2021-04-14

**Authors:** Praise Owoyemi, Sarah Salcone, Christopher King, Heejung Julie Kim, Kerry James Ressler, Ipsit Vihang Vahia

**Affiliations:** 1 Department of Psychology Univerity of California, Los Angeles Los Angeles, CA United States; 2 Department of Psychology University of South Alabama Mobile, AL United States; 3 Department of Psychology Emory University Atlanta, GA United States; 4 Division of Geriatric Psychiatry McLean Hospital Belmont, MA United States; 5 Division of Depression and Anxiety Disorders McLean Hospital Belmont, MA United States; 6 Department of Psychiatry Harvard Medical School Boston, MA United States

**Keywords:** electronic media, psychotherapy, text message, electronic mail, collateral information, telecommunication, communications media, digital

## Abstract

**Background:**

The review of collateral information is an essential component of patient care. Although this is standard practice, minimal research has been done to quantify collateral information collection and to understand how collateral information translates to clinical decision making. To address this, we developed and piloted a novel measure (the McLean Collateral Information and Clinical Actionability Scale [M-CICAS]) to evaluate the types and number of collateral sources viewed and the resulting actions made in a psychiatric setting.

**Objective:**

This study aims to test the feasibility of the M-CICAS, validate this measure against clinician notes via medical records, and evaluate whether reviewing a higher volume of collateral sources is associated with more clinical actions taken.

**Methods:**

For the M-CICAS, we developed a three-part instrument, focusing on measuring collateral sources reviewed, clinical actions taken, and shared decision making between the clinician and patient. To determine feasibility and preliminary validity, we piloted this measure among clinicians providing psychotherapy at McLean Hospital. These clinicians (n=7) completed the M-CICAS after individual clinical sessions with 89 distinct patient encounters. Scales were completed by clinicians only once for each patient during routine follow-up visits. After clinicians completed these scales, researchers conducted chart reviews by completing the M-CICAS using only the clinician’s corresponding note from that session. For the analyses, we generated summary scores for the number of collateral sources and clinical actions for each encounter. We examined Pearson correlation coefficients to assess interrater reliability between clinicians and chart reviewers, and simple univariate regression modeling followed by multilevel mixed effects regression modeling to test the relationship between collateral information accessed and clinical actions taken.

**Results:**

The study staff had high interrater reliability on the M-CICAS for the sources reviewed (*r*=0.98; *P*<.001) and actions taken (*r*=0.97; *P*<.001). Clinician and study staff ratings were moderately correlated and statistically significant on the M-CICAS summary scores for the sources viewed (*r*=0.24, *P*=.02 and *r*=0.25, *P*=.02, respectively). Univariate regression modeling with a two-tailed test demonstrated a significant association between collateral sources and clinical actions taken when clinicians completed the M-CICAS (β=.27; *t_87_*=2.47; *P*=.02). The multilevel fixed slopes random intercepts model confirmed a significant association even when accounting for clinician differences (β=.23; *t_57_*=2.13; *P*=.04).

**Conclusions:**

This pilot study established the feasibility and preliminary validity of the M-CICAS in assessing collateral sources and clinical decision making in psychiatry. This study also indicated that reviewing more collateral sources may lead to an increased number of clinical actions following a session.

## Introduction

### Background

Reviewing collateral information is an established practice in providing effective and targeted clinical care, particularly in psychiatry. Collateral information provides clinicians with critical information that they may not otherwise be able to obtain from patients’ self-reports. Although the importance of gathering collateral information is recognized [1,2], there has been little research of how it may be most effectively gathered, what types of information may be most informative, and how it impacts the clinical decision-making process. Although the gathering of collateral information is considered a part of routine care, this process merits closer study at this time, particularly with an exponential growth in available data from smart technology, wearable devices, and other sensors [3]. It is now feasible to use digital data to measure a broad range of neuropsychiatric symptoms, including depression, anxiety, insomnia, and apathy [4,5]. Similarly, with so much communication between individuals now happening digitally, there may be objective documentation of conversations that can be accessed as part of clinical care [6]. Thus, there is a need for a greater understanding of the types of information that are most relevant and impactful to care and clinical outcomes. This is especially relevant to psychiatry, where augmenting clinical assessment with digital and other data is beginning to impact care [7]. So far, no approach exists to quantify the types of information accessed by a mental health clinician during a typical visit. In addition to the electronic health record (EHR) review, there is no standardized approach or instrument to quantify or assess the full range of clinical decisions that may have been made during a typical clinical visit. EHR review has been shown to be an inadequate and inefficient approach for this because there is often no appropriate documentation of the entire decision-making process, and available information may be challenging to access systematically [8,9].

### Objectives

To further examine this issue, we developed an instrument that can measure the number of collateral information sources (CISs) that a clinician may have accessed during an individual session and the total number of clinical actions taken. In this study, we sought to pilot this instrument while also assessing how the nature and volume of collateral information (including digital data) collected may impact clinical decision making. Thus, this study has 2 primary aims. The first aim is to conduct initial feasibility testing of a new measurement tool, the McLean Collateral Information and Clinical Actionability Scale (M-CICAS), and establish both interrater reliability and preliminary validity of this measure against patients’ medical records. Our second aim is to test the hypothesis that accessing a greater amount of collateral information would be associated with a higher number of clinical actions taken by the participating clinician.

## Methods

### Developing the Measure

In developing our survey instrument, we sought to create a measure that could aggregate the number of sources of collateral information that the clinician accessed over the course of the session and determine which aspects of the clinical history the collateral information contributed to. Our approach was modeled on existing literature documenting the development of measures that were based on the aggregation of clinical actions [10,11]. We also aimed to quantify the number of clinical actions taken by the clinician during that session and determine how this information impacted clinical decision making as well as communication between the clinician and patient. To develop the items in the questionnaire, we adopted a consensus-based approach. As an initial step, the study principal investigator (IVV), in consultation with co-investigators, categorized the different types of collateral sources that may be accessed during clinical assessment and the clinical domains that may be impacted through the review of collateral information. On this basis, we selected the following 5 domains: (1) current clinical history, (2) past clinical history, (3) family history, (4) current functioning, and (5) current psychosocial status. Next, we used a similar consensus-based approach to list various clinical actions taken at the end of the session. We also consulted with clinicians who practiced in specialty psychiatry clinics (eg, geriatrics, child and adolescent, or substance use) to generate a more representative set of options. In addition, we requested input from peers at the University of Pennsylvania and Johns Hopkins University (listed in the Acknowledgments section) who are engaged in similar ongoing research.

### Final Survey Measure

The final survey consisted of 12 questions, divided into 3 sections (Multimedia Appendix 1). The first section asked clinicians what CISs they reviewed as part of the clinical session and then provided 11 concrete options as well as a write-in option for other sources. The 11 CIS options offered are as follows: review of medical records, labs, imaging, patient’s digital information, talk to mental health provider, talk to non–mental health provider, talk to family/caregiver, talk to nonfamily significant other, talk to patient’s school, talk to government agency, and patient-reported outcome measures. Checking off any of these options or the *other* option would prompt a question regarding whether the use of the checked-off collateral source provided additional information about the patient’s past or current clinical history, family history, functioning, or psychosocial status.

The second section of the survey sought to establish the clinical actions that were taken following the clinical session. Clinicians were asked whether they changed their treatment plan, adjusted the intensity of care, or took additional clinical actions. The additional clinical actions included changes regarding medications and/or psychotherapy modalities, calls, recommendations, referrals, clinical requests, and screenings. Finally, the third section of the survey examined the shared decision-making process between the clinician and the patient. Clinicians were asked whether they discussed alternatives, risks, side effects, and/or benefits of any of the treatment changes with their patients. This section was created to establish whether clinical actions are actually shared decisions between a clinician and patient, as there may be variability between practices, clinicians, and theoretical orientations. At the end of the survey, a general question was posed, asking clinicians to rate the extent to which they felt that accessing a patient’s electronic data might impact therapy outcomes. We established the reliability and validity of both parts distinctly. In the future, we anticipate that these can potentially serve as stand-alone instruments.

### Establishing Reliability

Our measure represents an aggregation of information sources accessed and clinical actions taken for a given session, and each score is specific to that session only. Thus, to establish reliability, we focused primarily on establishing interrater reliability using a common source of information (ie, EHR). We determined that establishing test-retest reliability would not be feasible, given the nature of this scale, because it was designed for cross-sectional assessment only.

### Demonstrating Validity

To demonstrate the validity of the M-CICAS, we elected to use the EHR as the *ground truth*. A primary driver for developing this measure was to quantify the sources of information reviewed and the actions taken during a session. We recognize that this approach may not be the most suitable because of multiple issues with the process of EHR documentation. The format of documentation in the EHR is nonstandardized, and different clinicians may apply different levels of detail. However, we determined by consensus that this may be the closest we can get to an objective standard by which to demonstrate the preliminary validity of our measure.

### Study Participants and Procedures

#### Description of Clinicians

We initially recruited clinicians at McLean Hospital in Belmont, Massachusetts, who provided ambulatory care to serve as study participants. The eligibility criteria included credentials to provide psychopharmacology or psychotherapy at McLean Hospital and licensed by the State of Massachusetts; actively practicing either adjunctive or stand-alone, evidence-based psychotherapy; and fluent in English. The study staff reviewed the procedures with eligible clinicians, and participation was voluntary. The study procedures were active, and data were collected over a period of 6 months. The participating clinicians completed the M-CICAS after an individual treatment session. Clinicians were asked to complete the measure after as many individual sessions as were feasible in their regular clinical schedule. Scales were completed by clinicians at follow-up visits with patients (ie, not at the intake or baseline assessments). For this study, a treatment session was defined as a single outpatient appointment providing evidence-based psychotherapy and/or pharmacotherapy. These outpatient sessions could also include family/caregivers/partners in the session as long as the patient was present.

After clinicians completed the M-CICAS, 2 members of the study staff (PO and SS) independently conducted a chart review using the clinical notes recorded by the participating clinician. The staff completed the M-CICAS using only the EHR note for the same encounter for which the clinician had completed the M-CICAS as the only source of information. Thus, we used 2 independently rated EHR-based versions of the M-CICAS to establish the reliability of the measure. Before completing the EHR-based data collection, the staff reviewed how to extract collateral information recorded in the medical record progress note before completing the chart reviews. To reduce potential bias, a separate study staff member entered clinician data, and the chart reviewers were blinded to the clinician data.

#### Testing Associations Between Collateral Information and Clinical Actions

As described earlier, this study has 2 primary goals. The first aim was to conduct feasibility testing of the M-CICAS, and the second was to assess whether there is an association between the amount of collateral information accessed and the number of actions taken by a clinician. For the 89 clinical encounters measured as part of this study, we assessed the associations between the number of data sources reviewed and clinical actions taken.

### Analytic Plan

A set of summary scores was generated for the number of collateral sources used in each clinical encounter and the total number of clinical actions taken following each clinical session. We then tabulated the clinician’s demographic data. Pearson correlations were used to determine the interrater reliability between clinicians and chart reviewers. To test the association between collateral sources reviewed and the number of clinical actions taken after a session, we first implemented simple univariate regression modeling without accounting for between-clinician differences. Given that subgroups of participants were nested within individual study clinicians and given that the heterogeneity of study clinicians could plausibly have an effect on the association between collateral sources reviewed and clinical actions taken, we also implemented multilevel mixed effects regression modeling. Simple univariate regression modeling was first applied to test the relationship between collateral sources accessed and the number of clinical actions taken. Next, multilevel mixed effects regression modeling evaluated whether significant variance in clinical actions taken could be attributed to interclinician differences. This allowed us to investigate whether interclinician differences explained significant variance in clinical actions taken and whether, when accounting for interclinician differences, there remained significant associations between collateral sources reviewed and the number of clinical actions taken. All statistical analyses were conducted using the statistical package R version 4.0.2 (R Foundation).

Study data were collected and managed using REDCap (Research Electronic Data Capture) electronic data capture tools hosted at the McLean Hospital [12]. REDCap is a secure, web-based application designed to support data capture for research studies, providing (1) an intuitive interface for validated data entry, (2) audit trails for tracking data manipulation and export procedures, (3) automated export procedures for seamless data downloads to common statistical packages, and (4) procedures for importing data from external sources.

## Results

### Clinician Demographics

Table 1 shows the breakdown of clinician demographic information. On average, clinicians reviewed 1.7 collateral sources per clinical appointment and conducted 1.36 clinical actions following each appointment. There was variability in the amount of survey data that each clinician completed. The range of the number of surveys completed by clinicians was broad, with one clinician completing surveys for 4 clinical sessions and another clinician completing surveys for 25 (mean 12.7, SD 8.3) clinical sessions. Overall, 3 clinicians worked primarily in geriatric settings (representing 42 encounters). The remaining 3 clinicians worked in general adult clinics (42 encounters) and 1 clinician in a child and adolescent clinic (5 encounters). Although we did not measure how long it took clinicians to complete the M-CICAS for each patient, based on the subjective impressions of clinicians 1 and 2, it was between 2 and 3 minutes per patient.

**Table 1:** Clinician demographics.

Clinician	Patient encounters, n (%)	Age (years)	Gender	Race	Clinical experience (years)	Professional degree	Therapeutic approach or approaches	Collateral sources viewed per patient, mean (SD)	Collateral actions taken per patient, mean (SD)
001	17 (19)	38	Female	White	8	APRN^a^	Cognitive behavioral therapy; supportive psychotherapy; mentalization and mindfulness-based therapies	1.35 (1.06)	1.71 (0.99)
002	21 (24)	41	Male	Asian	15	MD^b^	Psychodynamic; expressive therapy; supportive psychotherapy	2.14 (0.91)	1.67 (1.49)
003	25 (28)	30	Male	Asian	5	MD	Psychodynamic; mentalization and mindfulness-based therapies	1.88 (0.60)	0.76 (0.66)
004	4 (4)	69	Male	White	41	LMHC^c^	Cognitive behavioral therapy; acceptance and commitment therapy; supportive psychotherapy	0.25 (0.50)	0.5 (0.58)
005	9 (10)	46	Male	White	10	PsyD^d^	Cognitive behavioral therapy	1.78 (0.97)	1.44 (0.88)
006	8 (9)	65	Male	South Asian/Indian American	30	MD	Cognitive behavioral therapy; psychodynamic; supportive psychotherapy	0.5 (0.53)	0.88 (0.83)
007	5 (6)	31	Female	White	6	PhD^e^	Cognitive behavioral therapy; exposure therapy; dialectical behavioral therapy	4 (0.0)	2.6 (1.82)

^a^APRN: advanced practice registered nurse.

^b^MD: Doctor of Medicine.

^c^LMHC: licensed mental health counselor.

^d^PsyD: Doctor of Psychology.

^e^PhD: Doctor of Philosophy.

### Collateral Sources Viewed

Medical records were the most reviewed collateral sources of information (Table 2). Of the 89 clinical total encounters, 62 (70%) of the clinical sessions involved the clinician accessing medical records, distantly followed by 27 (30%) sessions reviewing information from another mental health provider. The remaining collateral source categories were accessed in less than 24% (21/89) of clinical visits. In 12% (11/89) of clinical appointments, the clinician reported not reviewing the collateral information.

**Table 2:** Type of collateral source reviewed (N=89).

Collateral source type	Percentage of surveys indicating review, n (%)
Medical records	70 (62)
Talk to mental health provider	30 (29)
Labs	24 (21)
Talk to family or caregiver	22 (20)
Patient reported outcome measures	11 (10)
Talk to patient's school	6 (5)
Other	4 (4)
Imaging	3 (3)
Patient's digital Information	2 (2)
Talk to non–mental health provider	2 (2)
Talk to nonfamily significant other	0 (0)

### Clinical Domains Impacted

Of the 89 total clinical encounters, 78 (88%) included a review of collateral information. Of the 78 visits where collateral information was reviewed, clinicians most frequently gained insight about a patient’s current clinical or mental status as a result of the review, as evidenced in 78% (61/78) of the visits. In 38% (30/78) visits, clinicians learned new information about a patient’s functioning through collateral review; in 33% (26/78) of visits, clinicians gained knowledge about their patient’s past clinical history; and in 31% (24/78) of visits, clinicians learned more about the patient’s psychosocial status. Finally, in 10% (8/78) of visits, clinicians gathered new information about the patient’s family history.

### Breakdown of Clinical Actions Taken

Clinicians reported adjusting patient medication after a session in 34% (30/89) surveys. Notably, clinicians wrote a separate action in the *other* category in 30% (27/89) of the responses. The remaining listed actions on the survey did not exceed 18% (16/89) of affirmative responses.

### Correlations Between Clinician Self-Report and Independent Staff Reviewers

Table 3 shows the correlations between ratings on the 2 sections of the M-CICAS by the 2 study raters and clinicians. Of note, although clinicians seemed to require only 2 to 3 minutes per patient to complete the M-CICAS, rater 1 reported requiring a mean of 3 minutes and 40 seconds (SD 1 min and 54 s) and rater 2 reported a mean of 3 minutes and 42 seconds (SD 1 min and 55 s). There was a range of 37 seconds for the shortest review to 9 minutes and 39 seconds for the longest per patient to review the EHR note and complete the scale for each visit. We found high interrater reliability between the study staff, both of whom independently completed their respective ratings and were blinded to clinician ratings (*r*=0.98, *P*<.001 between raters for sources viewed; *r*=0.97, *P*<.001 between raters for clinical actions taken). Comparisons between clinician ratings of CISs viewed (based on their self-report) and staff ratings (based on EHR review of the same visit) achieved moderate effect sizes and were also statistically significant (*r*=0.24, *P*=.02 and *r*=0.25, *P*=.02, respectively, between raters’ and clinicians’ ratings of sources viewed). However, the same comparisons on the clinical action subscale were not significant.

**Table 3:** Correlation matrix of study variables with significance level.^a^

Study Variable			1. Clinician source viewed		2. Clinician actions taken		3. Rater 1 sources viewed		4. Rater 1 actions taken		5. Rater 2 sources viewed		6. Rater 2 actions taken
**1. Clinician sources viewed**													
	*r*	—^b^											
	*P* value	—											
**2. Clinician actions taken**													
	*r*	0.26		—									
	*P* value	.02		—									
**3. Rater 1 sources viewed**													
	*r*	0.24		0.09		—							
	*P* value	.02		.41		—							
**4. Rater 1 actions taken**													
	*r*	0.06		0.11		0.32		—					
	*P* value	.55		.29		.002		—					
**5. Rater 2 sources viewed**													
	*r*	0.25		0.07		0.98		0.36		—			
	*P* value	.02		.53		<.001		<.001		—			
**6. Rater 2 actions taken**													
	*r*	0.06		0.13		0.30		0.97		0.34		—	
	*P* value	.58		.24		.004		<.001		.001		—	

^a^Relationship between number of information sources viewed and clinical actions taken.

^b^Not applicable.

Univariate regression modeling with a two-tailed test, not accounting for the clinician group, revealed a significant association between collateral sources reviewed and clinical actions taken when self-evaluated by clinicians (β=.27; *t_87_*=2.47; *P*=.02). To investigate whether this association was significant when accounting for clinicians, we first implemented multilevel random slopes and random intercept models. Analysis of variance tests indicated no significant differences in slopes between the clinician groups (*P*=.11). Consequently, we opted for a multilevel fixed slopes random intercepts model. Even when intercepts were allowed to vary by clinician within the model, there was a significant association between self-evaluated chart sources and clinician actions (Table 4).

**Table 4:** Clinician self-evaluated chart sources predicting clinician self-evaluated actions taken.

Predictors			Estimates		95% CI		*P* value
Intercept			0.04		−0.28 to 0.35		.83
Clinician sources viewed			0.24		0.02 to 0.45		.03
**Random effects**							
	Variance	0.85		N/A^a^		N/A	
	Between clinician variance	0.10		N/A		N/A	
	Intraclass correlation coefficient	0.11		N/A		N/A	
	N_clinician_	7		N/A		N/A	
	Observations	89		N/A		N/A	
	Marginal R^2^	0.056		N/A		N/A	
	Conditional R^2^	0.156		N/A		N/A	

^a^N/A: not applicable.

## Discussion

### Principal Findings

The primary goal of this study is to develop and conduct initial feasibility testing of a new two-part measure to quantify the number and types of sources of collateral information accessed by clinicians during a given session and the number of clinical actions taken during that session. We developed this measure based on input from several clinicians across 3 academic departments of psychiatry at McLean Hospital, the University of Pennsylvania, and Johns Hopkins University. We were able to demonstrate the feasibility of using this measure with 7 clinicians from a range of professional backgrounds across 89 patient encounters. Using chart review as the gold standard, we noted that the measure demonstrated acceptable validity (as measured by comparing clinician ratings during the session to staff rating based on chart review). We also noted that 2 independent staff raters had highly correlated scores on both sections of the M-CICAS when the staff raters scored it based on chart review, indicating acceptable reliability. Although the nature of the measure does not facilitate the demonstration of test-retest reliability or true construct validity, our approach is consistent with prior studies on similar measures [11].

We also noted a significant relationship between the number of sources of information reviewed and the number of actions taken (or treatment changes made) by clinicians. Although it is not clear whether a greater number of clinical actions leads to better clinical outcomes, this finding does point to the impact of collateral information on care.

Nonetheless, the implications of this finding are broader than those of this specific study. At a time when extraordinary amounts of information through digital sources are available to clinicians, a major undetermined question is whether access to this information may actually improve care [13]. Our findings provide an early signal suggesting that using collateral data from more diverse sources may have a positive impact on clinical care.

In a sense, our finding is consistent with a vast body of literature that suggests measurement-based care can improve outcomes [14,15]. However, we focused primarily on collateral information rather than quantifying symptom improvement. The M-CICAS may provide a way for researchers to focus on digital phenotyping and generate markers from the sources of digital data to differentiate which types of additional digital information are most relevant and impactful in clinical care. Thus, this scale may play a role in bridging the translation gap from proof-of-concept research on digital health into scaled implementation.

There are a number of limitations to our study. As this measure is an aggregation of the number of actions taken, the concept of construct validity is not applicable. Furthermore, because each scoring of the measure is applicable only to a single session, it was not possible to test true reliability except with the help of a chart review. Thus, our only gold standard to measure both validity and reliability involved reviewing progress notes of the session for which the clinicians completed the M-CICAS. This introduces the possibility of both recall bias and confounds from a lack of standardization in clinical documentation. Other methods to measure validity, such as audio recording or video recording of sessions or direct observation, may provide a higher level of objectivity; however, in this study, the logistical burden of these approaches was not feasible. In addition, as clinicians selected which patients they completed this survey with, the patient sample may not be representative. The relatively small sample size may also limit the generalizability of these findings. Finally, there was a broad range of completed surveys between individual clinicians, which may have introduced bias. Although we believe that our approach to analysis mitigates this effect, the impact of stylistic variations in clinical practice may remain. Nonetheless, we believe that as this measure is largely an aggregation of distinct actions taken by clinicians during a visit, the burden of establishing validity and reliability is lower because the measure is not intended to serve as a measure of abstract behavioral constructs.

### Conclusions

In summary, this study demonstrates the feasibility and utility and establishes baseline psychometric properties of the M-CICAS—a new measure that can quantify collateral information and clinical actionability in psychiatric care. Both these entities have been an integral element of clinical care for over a century, but their systematic measurement has not been a focus of research. This study also indicates that reviewing more sources of clinical information may be associated with greater amounts of clinical actions taken at a given session. When the availability of vast amounts of digital information places new burdens on clinicians, this measure may provide a way to determine what types of digital data are most relevant and impactful in patient care. As such, there has been very sparse research assessing how collateral information is collected and used. Our measure and this study represent only an initial step toward quantifying the collateral information used in clinical care. We intend for our approach to serve as a framework and expect that it may evolve to reflect new insights gained with broader application in more studies. We also anticipate that researchers and clinicians may adopt this scale to suit specific studies or clinical quality improvement projects. Our study also points to the possibility that reviewing more sources of clinical information may be associated with greater amounts of clinical actions taken at a given session, although this finding must be replicated in clinician and patient samples that are more standardized. Although this is a preliminary study that merits replication with larger representative samples, we believe that our approach may lay the foundation for a line of research that will facilitate more systematic translation of digital tools into psychiatric patient care.

## Appendix

Multimedia Appendix 1 McLean Collateral Information and Clinical Actionability Scale (M-CICAS).

## Abbreviations

CIS: collateral information source
EHR: electronic health record
M-CICAS: McLean Collateral Information and Clinical Actionability Scale
REDCap: Research Electronic Data Capture
