# Supplementary material for: Measuring and Quantifying Collateral Information in Psychiatry: Development and Preliminary Validation of the McLean Collateral Information and Clinical Actionability Scale
Source: JMIR Ment Health. 2021 Apr 14;8(4):e25050. doi: 10.2196/25050 (PMC8082386; doi:10.2196/25050)
Supplement: Multimedia Appendix 1 [file mental_v8i4e25050_app1.docx]

**McLean collateral information and clinical actionability scale (M-CICAS)**

1. Did you review/obtain collateral information from any of the following during today’s session? (Check all that apply)

[ ] Medical records [ ] Labs

[ ] Imaging [ ] Patient’s digital Information [ ] Talk to mental health provider [ ] Talk to non-mental health provider [ ] Talk to family/caregiver [ ] Talk to non-family significant other

[ ] Talk to patient’s school [ ] Talk to government agency (e.g. Child/Adult Protective Services, etc.) [ ] Patient reported outcome measures [ ] Other. Describe: [ ] None

(*branching logic for each option that is clicked, aside from none; “__” indicates a selected option from question 1)*

1. Did review of “____” allow you to gain any new information about the following? (Check all that apply)

[ ] Patient’s current clinical or mental status

[ ] Patient’s past clinical history

[ ] Patient’s family history

[ ] Patient’s functioning

[ ] Patient’s current psychosocial status

(*The next set of questions are not gated and will appear for all respondents*)

1. Has there been a change in your treatment plan after today’s session?

[ ] Yes [ ] No

1. Did you change (increase/decrease) intensity of care (including the visit frequency, number of visits, etc.)?

[ ] Increase [ ] No change [ ] Decrease

1. After today’s session, did you take any of the following actions:

[ ] Adjust patient’s medication (increase, decrease, add, or discontinue)

[ ] Adjust patient’s psychotherapy plan/modality

[ ] Schedule phone call between sessions

[ ] Plan to bring additional person to next session (e.g. parent, caregiver, partner, etc.)

[ ] Recommend any use of apps between sessions

[ ] N/A

[ ] Other. Describe: _____________

1. After today’s session, did you refer to any of the following:

[ ] Higher level of care (Inpatient, Urgent Care, etc.)

[ ] Mental health professional (Individual or Group)

[ ] Medical Provider (Primary Care Provider, Neurologist, Internist, etc.)

[ ] Exercise or nutritional program

[ ] Support group

[ ] N/A

[ ] Other. Describe: _____________

1. After today’s session, did you request any of the following:

[ ] Labs

[ ] Imaging

[ ] Neuropsychological testing

[ ] N/A

[ ] Other. Describe: _____________

1. After today’s session, did you call any of the following:

[ ] Patient’s school

[ ] Government agency

[ ] Patient’s parent/caregiver or partner

[ ] N/A

[ ] Other. Describe: _____________

(*branching logic: the following 3 questions will appear if question 3 is marked as yes)*

1. Were alternatives to the change in treatment plan discussed?

[ ] Yes [ ] No

1. Were the risks and benefits of the treatment change discussed?

[ ] Yes [ ] No

1. Were any potential side effects of the treatment change discussed?

[ ] Yes [ ] No
